# Growth Performance and Biochemical Composition of Black Soldier Fly Larvae (*Hermetia illucens*) Reared on Diets Containing Similar Crude Protein Content

**DOI:** 10.3390/insects17050504

**Published:** 2026-05-15

**Authors:** Somaya Naser El Deen, Klaas van Rozen, Hellen Elissen, Piet van Wikselaar, István Fodor, Roomie van der Weide, Elise Hoek-van den Hil, Arya Rezaei Far, Teun Veldkamp

**Affiliations:** 1 Wageningen Livestock Research, De Elst 1, 6708 WD Wageningen, The Netherlands; piet.vanwikselaar@wur.nl (P.v.W.); istvanfodor.dvm@gmail.com (I.F.); arya.rezaeifar@wur.nl (A.R.F.); teun.veldkamp@wur.nl (T.V.); 2 Wageningen Plant Research, Edelhertweg 1, 8219 PH Lelystad, The Netherlands; klaas.vanrozen@wur.nl (K.v.R.); hellen.elissen@wur.nl (H.E.); rommie.vanderweide@wur.nl (R.v.d.W.); 3 Wageningen Food Safety Research, Akkermaalsbos 2, 6708 WB Wageningen, The Netherlands; elise.hoek@wur.nl

**Keywords:** circularity, waste management, unauthorised diets, correlations

## Abstract

For optimal growth and development, black soldier fly larvae require a balanced diet. This study focused on how nutrients other than protein, specifically fat, starch, fibre, and ash, affect larval growth, body composition, and the quality of the leftover material called frass. To isolate the effects of these nutrients, five types of organic byproducts: fast food (FF) waste, solid pig manure (PS), mushroom stems (MS), slaughter waste (SW) and poultry meal (PM), were used to create six different diets, all with similar protein levels (about 22% DM). The results showed that diets rich in fat and starch, such as those based on FF waste, produced the heaviest larvae (155.9 mg), which also had high fat (30.31% DM) and protein contents (52.74% DM). In contrast, diets based on PM, which were low in fat and starch but high in fibre and ash, resulted in lower larval weight, which had lower fat content but variable protein content depending on other diet ingredients. Similar dietary protein levels yielded different larval protein contents, indicating that other nutrients may have influenced how protein was stored. However, other components of the diet, especially fat and ash, were consistently reflected in both the larvae and the frass. Overall, the study shows that nutrients other than protein play important roles in larval development and should be considered when designing black soldier fly diets.

## Simple Summary

Black soldier fly larvae are used to turn food and farm byproducts into useful products, but they need the right mix of nutrients to grow well. This study looked at how different nutrients, such as fat, starch, and fibre, affect the growth and body composition of the larvae, as well as the quality of the leftover material called frass, which is an organic fertiliser. Six diets were created using different types of byproducts, all with similar protein levels, to see how other nutrients influenced the results. Results show that diets rich in fat and starch produced larger larvae with more body fat and protein, while diets with more fibre and ash led to smaller larvae with less fat. The protein in the diet did not always end up in the larvae, showing that other nutrients also play a role. The waste left behind by the larvae also reflected the nutrients in the diet, especially fat and ash, but not nitrogen. These findings help us better understand how to feed the larvae in ways that improve both their growth and the quality of the fertiliser they produce, which can benefit sustainable farming and waste recycling.

## 1. Introduction

Over the past decade, interest in the mass rearing of several detritivore insect species (or their larvae) has increased [1]. Several reasons lie behind this interest, such as the vast amount of organic waste produced, the waste-processing capabilities of these insects and the rising prices of animal feed products. Agriculture, agro-industrial processes, restaurants, and households generated around 931 million tons of organic byproducts and food waste in 2019 (17% of the total global food production) [2]. Detritivore insects can be reared on low-grade biowastes and convert them into high-quality proteins, lipids and other useful products. In addition, the scarcity of resources has led to increased prices of animal feedstock, which represents 60% or more of the costs of animal production systems [3,4]. Black soldier fly, BSF, *Hermetia illucens* L. (Diptera: Stratiomyidae) larvae are excellent candidates for use as animal feed. The advantages of using BSF larvae (BSFL) are their capacity to convert organic waste, reduce the numbers of certain harmful bacteria and insect pests, provide potential chemical precursors to produce biodiesel, and supply high-quality protein to be used as feed for animals, including swine, poultry and fish [5,6].

The choice of diet for BSFL during the rearing requires a careful balance between meeting the larvae’s biological needs and achieving efficient bioconversion, while also ensuring cost-effective, sustainable production within safety and legal boundaries. One of the most important factors affecting BSFL growth rates is diet quality [7]. The larval survival rate tends to increase with high-quality diets (rich in protein and fat), whereas the duration of the larval stage tends to decrease [8,9,10]. High-quality diets also increase BSFL weight and size [10,11,12]. Moreover, diet quality influences the larval body composition [10,11]. Macronutrients [13] and, to a lesser extent, micronutrients [13,14] can affect larval composition. Many studies have investigated the effect of diets, with varying nutritional compositions and formulated with a wide range of byproducts and side streams, on the BSFL development and composition [15,16,17,18]. Across different diets, larval protein content has ranged between 37.0% and 62.7% on a dry matter (DM) basis and fat content ranged between 6.6% and 39.2% DM [13]. Larval fatty acid content also varied with different diets [14,19,20]. Barragan-Fonseca (2017) found that larvae fed good-quality diets may require less feed to achieve good performance than larvae fed low-quality diets [13]. For animal feed applications, producing insects of defined quality is essential; however, different biotic and abiotic conditions (e.g., larval density, substrate and air humidity, and other system characteristics) can affect larval performance and must be considered when comparing different studies [21,22].

Different diets vary in their contents of protein, fat, starch, fibre, and a long list of micronutrients, which complicates the attribution of the individual effects of these macro- or micronutrients on BSFL traits. We hypothesise that macronutrients other than protein affect BSFL growth performance and biochemical composition, as well as frass composition. To test this hypothesis, the current study analysed the effects of six different diets with roughly similar crude protein contents (~22% DM) on BSFL growth and development. In addition, the larval crude protein and fat contents (including fatty acid composition), conversion efficiencies, nutrient losses and the biochemical composition of the frass were measured and analysed across diets. This design should eliminate the effect of diets’ crude protein content and clarify the effects of the other macronutrients. To achieve this objective, five organic and industrial waste streams were selected: fast food (FF) waste, solid pig manure (PS), mushroom stems (MS), slaughter waste (SW) and poultry meal (PM). These streams were used to formulate six diets with comparable crude protein content. Although not all selected streams are currently legal for use in the EU, this study highlights their potential as feeding substrates for BSFL. The safety of BSFL reared on these diets was assessed [23], and the results will be presented to EU policymakers. The outcomes of this experiment can contribute to circular-economy goals by enabling more efficient reduction and/or reuse of organic wastes. Moreover, this study may identify promising diet mixtures that enhance BSFL production.

## 2. Materials and Methods

### 2.1. Diet Preparation

Six diets were formulated from five biowaste streams: fast food (FF) waste, solid pig manure (PS), mushroom stems (MS), slaughter waste (SW) and poultry meal (PM). The fast food waste consisted of fries, vegetables, bread, and meat products. Solid pig manure was sow faeces. Mushroom stems (*Agaricus bisporus*) included a small amount of residual growing substrate remaining after harvesting mushrooms. Slaughter waste was the solid component of the secondary sludge generated at a slaughter wastewater treatment facility. The poultry meal was a commercial protein source processed from poultry byproducts and commonly used in dry pet food production. All streams were obtained one week before the start of the rearing cycle and stored at 4 °C until use. On arrival, the mushroom stems were pre-treated in a bowl cutter (Mado Supra 50, Dornhan, Germany) to a particle size of ~0.5 cm and the other ingredients were homogenised with a drill mixer (Vonroc, Zwolle, The Netherlands). After preparation, each ingredient was sampled and analysed for DM, crude protein and fat by an ISO 9001:2026-certified laboratory [24]. The results are presented in Table 1.

The diets were formulated with different amounts of each waste stream/ingredient (Table 2) to achieve comparable crude protein (~22% DM) and dry matter (30%) contents. Diets were prepared one day before the start of the experiment by mixing the ingredients in barrels with a drill mixer. The diets were kept overnight at room temperature with the barrels covered to limit water loss.

Cellulose and water were used to adjust diet dry matter (DM) (Table 2). Cellulose was added to increase DM and water to decrease it, standardising all diets at 30% DM. Cellulose (Alphacel Non-Nutritive Bulk, MP Biomedicals, Irvine, CA, USA) is considered indigestible for BSFL due to the larvae’s low cellulase digestive activity [25,26].

### 2.2. Experimental Setup

Black soldier fly larvae (BSFL, *Hermetia illucens*) were obtained from the commercial producer company Bestico (Berkel en Rodenrijs, The Netherlands). On arrival, the larvae were 5 days old and had been reared on the company’s standard BSFL diet. The larvae were sieved through a 2 mm mesh to remove residual substrate; residual frass from the source diet was negligible after sieving. The mean starting individual fresh weight was 11.98 mg, determined from five samples of 200 larvae each.

Plastic containers (75 × 47 × 15 cm) were each filled with 10 kg of diet in a ~ 5 cm deep layer. Each container received 1850 larvae per one kg fresh diet, equivalent to 0.54 g diet per larva over 7 days (internal protocol). The number of starter larvae was determined by mass-based counting using the average individual larval weight (1850 × 11.98 mg = 22.16 g larvae per 1 kg fresh diet).

The container was the experimental unit. Each of the six diets was replicated three times (i.e., *n* = 3 per diet), resulting in 18 containers in total. The containers were stacked in three columns in a climate chamber, with each stack containing six containers (one container per diet). Containers corresponding to the highest fat diets were placed at the bottom to minimise cross-contamination from any escaping larvae.

The chamber temperature was maintained at 30.0 ± 1.0 °C throughout the experiment. Relative humidity (RH) was kept ≥70% on days 1 and 2, increased to 90% on days 3 and 4 to compensate for evaporation losses due to larval activity, returned to 70% on day 5, and lowered to 60% on day 7 (last day). During the whole experiment, the rearing chamber was kept dark (0:24 L:D), with only indirect illumination from a monitoring camera.

### 2.3. Measured Parameters

Before the experiment, 54 diet samples (~0.6 kg each) were collected for biochemical analysis (triplicate per container; nine samples per diet).

After 7 days of incubation, containers were weighed, and larvae were manually sieved to separate them from frass. Frass was weighed, and 3 samples were collected from each container for biochemical analysis (54 samples in total). From each container, three samples of larvae (about 50–100 larvae per sample) were taken, counted and weighed.

The biochemical composition of the diets, larvae, and frass (moisture/water content, crude ash, crude protein (N × 6.25), total fat, and crude fibre) was analysed by AGROLAB LUFA GmbH (Kiel, Germany) using the official sampling and analytical methods described in Commission Regulation (EC) No. 152/2009 of 27 January 2009 [27]. Larval fatty acid content was analysed following protocols accredited by the German Accreditation Body using gas chromatography with flame ionisation detection (GC-FID) for quantitative determination, and certified FAME standard mixtures for identification and quantification.

Larval growth rate (mg/d) was calculated by the following equation:

GRlarvae (mg/d) = (LWfinal − LWinitial)/d(1)

To evaluate larval efficiency in consuming and metabolising the diets, the total final biomass (larvae + prepupae) and residual diet were weighed. The waste reduction index (WRI) and the conversion efficiency (ECI) were calculated on a DM basis.

The following indices were calculated:

WRI = ((S − F)/S)/d(2)

ECI = LWgain/(S − F)(3)

CP mass larvae (g) = LWfinal × CPlarvae(4)

G mass larvae (g) = LWfinal × Glarvae(5)

DMconversion (%) = LWgain/S × 100(6)

CPconversion (%) = CPlarval gain/CPS × 100(7)

Gconversion (%) = Glarval gain/GS × 100(8)

DMloss (%) = (S − LWgain − F)/S × 100(9)

CPloss (%) = (CPS − CPlarval gain − CPF)/CPS × 100(10)

Gloss (%) = (GS − Glarval gain − GF)/GS × 100(11)

Calculation of the chemical composition parameters:

Content of macronutrients on a dry matter basis (%):

Macronutrient % in DM = Macronutrient % in fresh medium/DM content (%) × 100(12)

Content of individual fatty acids (FA) on dry matter basis (%):

FA (%) = FAG × G/100(13)

GR: growth rate; LW: larval weight (DM); d: days; S: starting diet (DM); F: frass (DM); G: total fat (DM); CP: crude protein (DM); DM: dry matter; FA: fatty acid.

### 2.4. Statistical Analyses

Each response variable was analysed by two-way ANOVA with diet and stack as fixed factors. Model diagnostics were performed by visual inspection of residual plots. Pairwise comparisons between diets were performed using Tukey’s post hoc test via the multcomp package in R version 4.1.0 [28]. Values below the detection limit were treated as missing data. For each response, only diets with complete measurements were included. The significance level was set at α = 5%. Relationships between nutritive compounds in diet, larvae, and frass were analysed using Kendall’s rank correlation. Kendall’s tau (τ) ranges from −1 (perfect negative correlation) to 1 (perfect positive correlation), with τ = 0 indicating no correlation. Compounds with complete measurements for at least five diets qualified for correlation analysis. Statistical analyses were performed in R version 4.1.0 (R Core Team, 2021).

## 3. Results

### 3.1. Diet Biochemical Composition and Fatty Acid Content

The analysis confirmed that the crude protein content of all diets was similar (~22% DM, *p* = 0.0584). DM was also comparable (~30%), with FF showing the highest DM (30.8%) and PS-MS-PM having the lowest (28.2%). Other components (ash, total fat, crude fibre, and starch) were significantly different among diets (Table 3).

The FF diet had by far the highest fat (31.2%) and starch (29.3%) contents compared to all other diets. Its fat content was 5.9-fold higher than that of the lowest fat-content diet, PS-MS-PM (5.3%). Starch in PS-MS-PM and PS-MS-SW diets was below the detection limit. In contrast, the PS-MS-PM diet had the highest crude ash (12.5%) and crude fibre (32.9%), whereas the FF diet had the lowest values (crude ash: 3.7%; crude fibre: 6.5%).

For minerals, the PS-MS-PM diet contained the highest amount of potassium (1.0%), calcium (2.5%) and phosphorus (1.3%), while FF had the lowest concentrations. Diet pH ranged from acidic (4.5 in FF) to near neutral (7.4 in PS-MS-PM) (Table 3).

The fatty acid composition of the diets was analysed, and results are presented in Table 4 and Appendix A. Significant differences were observed among diets for most fatty acids. FF and FF-MS-SW diets showed the highest concentrations of myristic acid, palmitic acid, omega-6 and the sum of saturated fatty acids. Lauric acid was highest in FF-MS-SW and PS-MS-SW diets. The FF diet had the greatest amounts of mono- and polyunsaturated fatty acids and omega-3 among other diets. Capric acid and the sum of trans fatty acids did not differ significantly among diets. Overall, PS-MS-PM had the lowest fatty acid content compared to other diets.

### 3.2. Larval Growth Performance, Waste Reduction, and Conversion Efficiency

**Figure 1:**
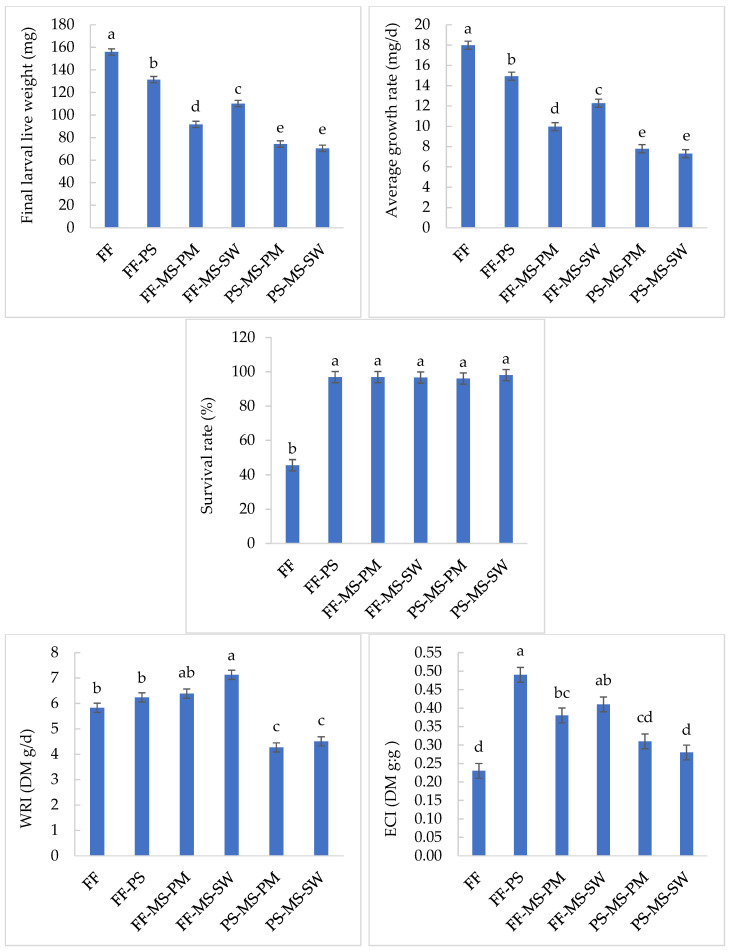
Mean values ± SEM of final live weight (mg), average growth rate (mg/d), survival rate (%), waste reduction index (DM g/d) and efficiency of conversion of the ingested diet (DM g:g) of BSFL grown on different diets. Means with different superscript letters differ significantly (two-way ANOVA followed by Tukey’s post hoc test analysis, *p* < 0.05). Fast food (FF), pig manure solid (PS), mushroom stems (MS), slaughter waste (SW) and poultry meal (PM).

On harvest day, no larvae had escaped. Diets had significantly affected larval growth, waste reduction and diet conversion (Figure 1). On FF, larvae reached the highest live weight (155.9 mg) and average growth rate (18.0 mg/d), but survival was the lowest (45%) among diets. PS-MS-PM and PS-MS-SW diets had the lowest larval weight and average growth rate. The highest WRI was observed on the FF-MS-SW diet (7.1 g/d, DM), which was 1.2 times higher than the FF diet (5.8 g/d) and 1.7 times higher than the lowest WRI value (4.3 g/d) measured for the PS-MS-PM diet. The Efficiency of Conversion of Ingested diet (ECI) was highest on the FF-PS diet (0.5), followed by FF-MS-SW (0.4), which did not differ significantly. The lowest ECI was recorded on FF (0.23) and PS-MS-SW diet (0.28). These four parameters (live weight, growth rate, WRI, and ECI) did not differ significantly between PS-MS-PM and PS-MS-SW.

### 3.3. Larval Biochemical Composition and Fatty Acid Content

**Figure 2:**
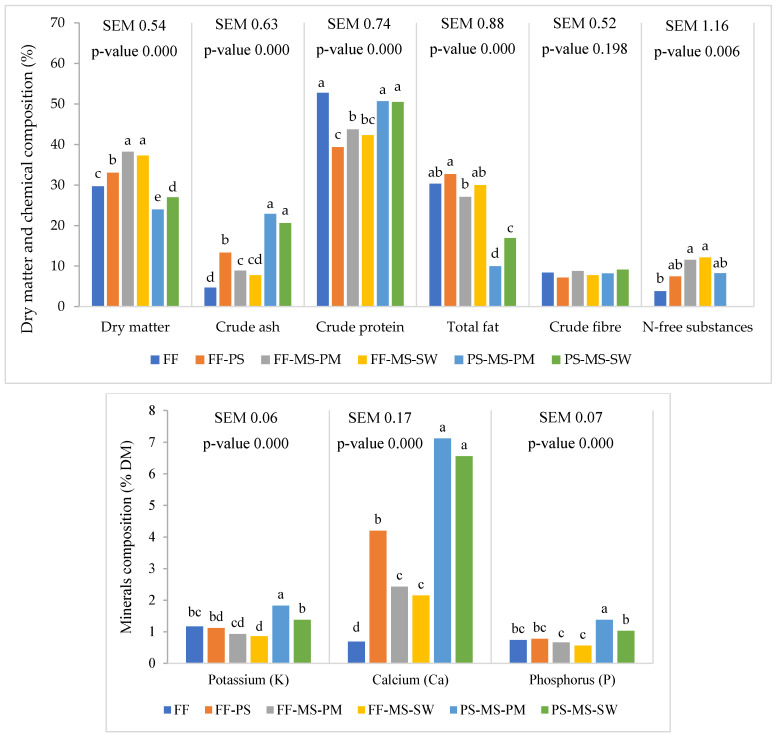
Mean values of DM and biochemical composition of larvae reared on different diets (DM expressed in % of fresh weight, all nutrients expressed in % on DM basis). For each nutrient, means with different superscript letters differ significantly (two-way ANOVA followed by Tukey’s post hoc test analysis, *p* < 0.05). Fast food waste (FF), solid pig manure (PS), mushroom stems (MS), slaughter waste (SW) and poultry meal (PM).

Larval biochemical composition was analysed, and results are presented in Figure 2.

Larvae reared on FF-MS-PM and FF-MS-SW had the highest DM (38.2 and 37.3% respectively). The lowest larval DM content was observed for the PS-MS-PM diet (24.0%), 1.6-fold lower than the highest values. Larval crude protein was the highest on both PS-based diets and the FF diet. However, the FF-PS diet yielded the lowest larval crude protein content (39.4%); 25.4% lower than on FF alone (52.7%). The highest larval fat content occurred on the FF-PS diet (32.7%), which was similar to other FF-based diets but higher than on FF-MS-PM. Larvae reared on the PS-MS-PM diet had by far the lowest fat content (10.0%), 3.3-fold lower than the highest value on FF-PS.

Potassium, calcium and phosphorus showed the same trend, with the greatest accumulation in larvae reared on PS-MS-PM. Calcium content was also significantly higher in larvae fed PS-MS-SW.

The larval fatty acid composition was analysed, and the results are presented in Table 5 and Appendix A. Diets significantly affected the concentrations of several fatty acids in larvae but not the sum of polyunsaturated or trans fatty acids. Overall, larvae reared on FF-based diets had significantly higher concentrations of fatty acids. Capric acid and the sum of monounsaturated fatty acids were highest in FF-fed larvae. Lauric acid, myristic acid, and the sum of saturated fatty acids were highest in larvae on FF-PS. Larvae reared on PS-MS-PM had the lowest palmitic acid. Larvae reared on all the FF-based diets had the highest omega-3 and 6 fatty acid concentrations.

### 3.4. Larval Dry Matter, Crude Protein and Fat Masses, Conversions and Losses

**Figure 3:**
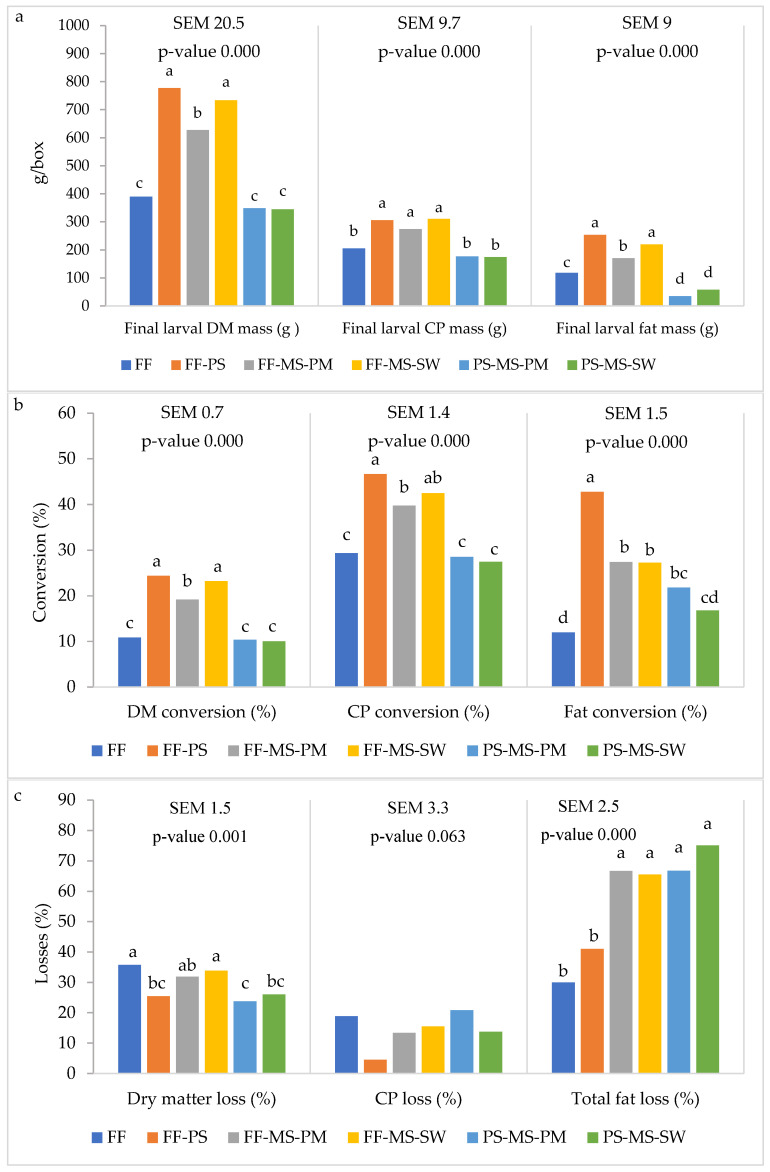
Mean values of DM, crude protein and fat masses (g/box) (**a**), conversions (%) (**b**) and losses (%) (**c**) of larvae reared on different diets (based on dry matter). Means with different letters are significantly different (two-way ANOVA followed by Tukey’s post hoc test analysis, *p* < 0.05). Fast food (FF), pig manure solid (PS), mushroom stems (MS), slaughter waste (SW) and poultry meal (PM).

Figure 3 presents larval dry-matter (DM), crude protein (CP), and fat masses, and the associated conversions and losses (DM basis). All parameters differed significantly among diets.

Across diets, the final larval DM, CP, and fat masses were highest on FF-PS and FF-MS-PM diets, and lowest on FF, PS-MS-PM and PS-MS-SW. Reared on FF-MS-PM, the larvae also had significantly high CP mass (274.16 g).

Dry matter conversion was highest on FF-PS (24.4%) and FF-MS-SW (23.2%), and the lowest on PS-based diets and the FF diet. Similarly, CP conversion was the highest on FF-PS (46.6%), followed by FF-MS-SW (42.5%), with no significant difference. The lowest values were observed for PS-MS-SW, PS-MS-PM and FF diets. Fat conversion was highest on FF-PS (42.8%) and lowest on FF (12.0%), 3.6-fold lower.

DM loss was highest on FF (35.8%) and FF-MS-SW (33.9%), similar to FF-MS-PM, and lowest on PS-MS-PM diet (23.8%). Fat losses were lowest on FF (30.0%) and FF–PS (40.0%) compared with the other diets.

### 3.5. Frass Biochemical Composition and pH

**Figure 4:**
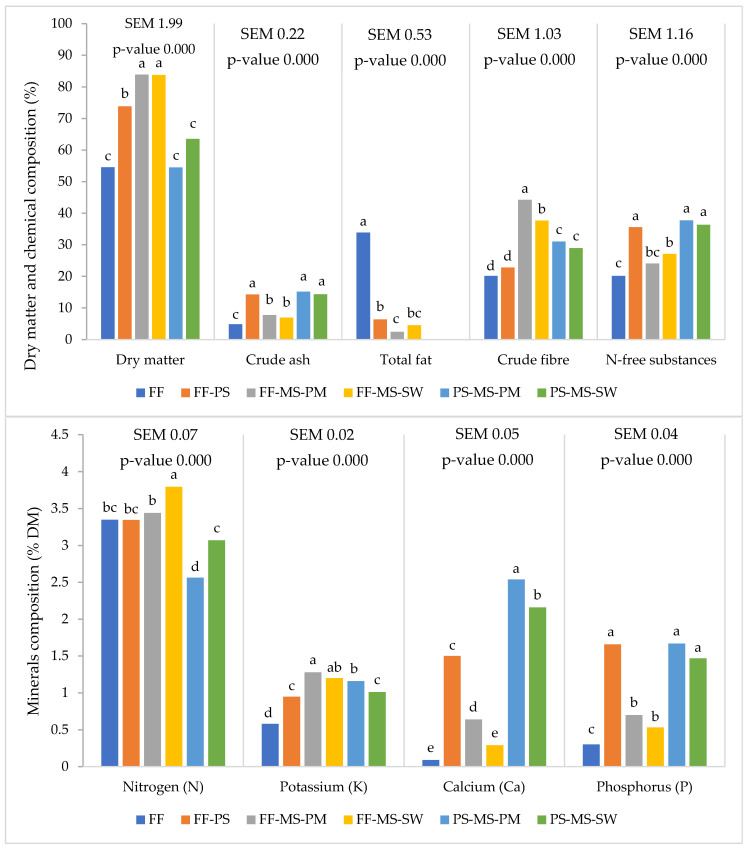
The mean values of dry matter and biochemical composition of frass of different diets (dry matter expressed in %, all nutrients expressed in % of dry matter). For each nutrient, means with different superscript letters differ significantly (two-way ANOVA followed by Tukey’s post hoc test analysis, *p* < 0.05). Fast food waste (FF), solid pig manure (PS), mushroom stems (MS), slaughter waste (SW) and poultry meal (PM).

Different diets affected the biochemical composition of frass (Figure 4). Dry matter (DM) was highest in frass from FF–MS–PM (83.9%) and FF–MS–SW (83.8%). Ash content was highest in frass from the three PS-based diets and lowest in FF frass (4.8%). Total fat was by far highest in FF frass (33.9%) and lowest in FF–MS–PM (2.5%). Crude fibre was highest in FF–MS–PM frass (44.2%) and lowest in FF frass.

The N, P, K and Ca contents of the frass also varied by diet (Figure 4). Nitrogen was highest in FF–MS–SW frass (3.8%), 1.5-fold higher than the lowest value on PS–MS–PM (2.6%). Potassium was highest in FF–MS–PM frass (1.3%). Calcium was highest in PS–MS–PM frass, whereas phosphorus was highest across all PS-based diets. For P, K, and Ca, the lowest concentrations occurred in FF frass.

Frass pH ranged from acidic to basic. PS–MS–PM produced basic frass (pH 8.3), whereas FF produced acidic frass (pH 6.0). The remaining diets yielded near-neutral frass (pH 6.9–7.7).

### 3.6. Correlation Biochemical Compositions Diet–Larvae–Frass

The diet–larvae–frass composition is an intercorrelated complex system, and the composition variability of one (e.g., diet) will affect the composition of the others (e.g., larvae and frass).

We found strong correlations between the biochemical composition of the diet and the larvae. The diet’s DM content was negatively correlated with larval ash content (τ = −0.53, *p* = 0.0027). Larvae reared on diets with high ash content had high ash content as well (τ = 0.82, *p* < 0.0001), but low total fat (τ = −0.47, *p* = 0.0064). Fat content in the diet was positively correlated with larval fat (τ = 0.54, *p* = 0.0019) and larval DM (τ = 0.40, *p* = 0.0207) content, but a negative correlation was found with larval ash content (τ = −0.78, *p* < 0.0001). Larvae reared on high fibre diets had low fat (τ = −0.71, *p* < 0.0001) and high crude ash (τ = 0.59, *p* = 0.0006) contents. However, the crude protein content of the diet was not strongly correlated with any of the larval macronutrients (all |τ| ≤ 0.6). Regarding the fatty acid profile, most of the fatty acids in the diets were moderately or strongly and positively correlated with the fatty acids in the larvae (τ ≥ 0.30) (Appendix B).

In general, the correlations between the composition of the diet and frass were weaker than those observed between the diet and larvae. Higher ash content in the diet was correlated with higher ash (τ = 0.77, *p* < 0.0001), but lower crude protein content (τ = −0.54, *p* = 0.0019) in the frass. The pH of the frass was also higher when larvae were reared on an ash-rich diet (τ = 0.71, *p* < 0.0001). Larvae reared on diets with higher fat content left behind frass rich in N (τ = 0.55, *p* = 0.0015), but poor in ash (τ = −0.73, *p* < 0.0001) and of low pH (τ = −0.72, *p* < 0.0001). The crude protein content in the diets was not strongly correlated to any of the frass macronutrients (all |τ| ≤ 0.6).

## 4. Discussion

This study evaluated the growth performance and the biochemical composition of BSFL reared on six different diets with roughly similar crude protein content (~22% DM). The frass biochemical composition was also analysed.

The larval DM differed significantly, ranging from 24.0% to 38.2% of fresh weight. Substrate composition, larval stage and larval fat content can all affect DM accumulation [29,30]. Eriksen (2022) reported an inverse relationship between larval water and lipid contents [29]. Our results align with this because larval DM was positively correlated with dietary fat (τ = 0.63), as adipose tissues bind less water [31].

In this study, FF-based diets (FF, FF-MS-SW and FF-PS) yielded the best larvae. FF-based diets showed the highest values for final larval live weight (FF; 155.9 mg), average growth rate (FF; 18.0 mg/d), WRI (FF-MS-SW; 7.13 g/d) and ECI (FF-PS; 0.5). In the current research, the larval performance was different between diets despite similar CP, but different fat (5.3–31.2%) and starch (11.5–29.3%; absent in PS-based diets). Therefore, the BSFL growth responds to different dietary nutrients such as fat and carbohydrates and not only to crude protein. Likewise, Barragan-Fonseca et al. (2019) found that larval yield was highest on diets rich in carbohydrates, independent of protein level [32]. Larvae reared on PS-based diets had the lowest live weight, growth rate, WRI and ECI, similar to other results using pig manure as a diet [15,33]. This likely reflects insufficient readily available energy and nutrients in the pig manure, combined with a high fibre content that reduces digestibility and digestive efficiency for BSF larvae.

Larval crude protein content was highest on the FF diet (52.7%) and PS-based diets (50.7 and 50.5%), and the lowest on the FF-PS diet (39.4%), despite similar dietary CP. These results partly agree with Naser El Deen et al. (2023) [33] results. In that study, larvae grown on fast food waste, composed of fries, vegetables, bread, and meat products (18.1% CP and 27.7% fat), produced the best performance but the lowest larval CP (38.5%), unlike our FF outcome. On the other hand, the findings of the current study are consistent with those of Nguyen et al. (2015) [11].

In their study, larvae reared on kitchen waste (plant and animal material; 20.4% CP) had significantly higher larval weight and length, and the highest CP (21.2%) [11], which is also the case for the FF diet in this study. Together, these results show that a CP level around ~22% in the diet supports maximum larval protein deposition.

PS-based diets (PS-MS-PM and PS-MS-SW) also produced high larval CP, which can be explained in different ways. First, like other diets, PS-based diets contained ~21% CP, which is the necessary amount for larval protein deposition as mentioned above. Second, BSFL reared on PS-based diets showed the lowest growth performance, as reflected in reduced live weight and growth rate at harvest. However, lower larval mass does not necessarily correspond to an earlier instar stage. Barragan-Fonseca et al. (2019) demonstrated that larvae fed low-quality diets (characterised by low protein and carbohydrate content or low protein alone) progress more rapidly to the prepupal stage, resulting in a shorter overall development time. This suggests that BSFL may trade off body size for faster development [32]. Liu et al. (2017) reported that crude protein content decreases during mid-larval development and increases again during the pupal stage [30]. In our study, larvae were harvested in the prepupal stage, close to the point of maximal protein deposition.

PS-based diets were also rich in fibre, which may have influenced the larval CP. Some studies showed positive effects of fibre-rich diets on BSF larval CP [34,35,36], whereas another study found the lowest larval CP on fibre-rich (lignin) substrates [37]. In the current study, the inclusion of cellulose varied across diets to standardise the dry matter. Although cellulose is a nutritionally inert material that may not impact the larval biochemical composition, it can alter the diet’s physical characteristics, and thus the larval feeding process, larval growth, survival rate and weight [38]. Therefore, the effects of fibre on the growth and composition of BSFL should be further studied to clarify this.

The larval crude protein differed across diets despite the roughly similar crude protein content (~22%). This confirms that diet CP does not solely determine the larval CP, and only a small fraction of larval CP variation could be explained by dietary protein [32,39,40]. In Naser EL Deen et al. (2023), both diets with the highest and lowest CP produced crude protein-rich larvae [33].

The correlation analysis confirms the above-mentioned results. Our results showed that the crude protein in the diet did not have a strong correlation with the larval crude protein content. This suggests that the amount of protein the larvae can assimilate from the diet is relatively fixed, and a protein-rich diet may not necessarily yield protein-rich larvae, as also shown by Nguyen et al. (2015) [11]. In the latest study, BSF larvae were reared on diverse substrates and nutritional compositions (poultry feed, pig liver, pig manure, kitchen waste, fruits and vegetables, and fish rendering). Larvae reared on pig liver and kitchen waste had similar crude protein content (21%), even though pig liver had far higher crude protein (76.71%) than kitchen waste (20.41%).

Beyond all the factors mentioned above that affect the crude protein content in BSFL, using the standard nitrogen-to-protein conversion factor (6.25) may overestimate the BSFL crude protein content due to chitin and other non-protein nitrogenous compounds (e.g., nucleic acids, uric acid, urea, and ammonia) [41]. Additionally, diets with similar CP can differ in amino acid profiles important for BSFL growth. Therefore, additional studies are needed to determine the roles of different amino acids on BSFL performance and nutritional composition.

Total fat was significantly higher in larvae reared on FF-based diets than on PS-based diets. In addition to the higher dietary fat, FF-based diets also had high starch content. Both dietary fat and starch likely contributed to fat accumulation, resulting in fat-rich larvae, as shown in other studies [39,42]. In the literature, high larval fat is attributed to a high dietary carbohydrate content, independent of the protein content [32,43]. The larval fat content on FF-based diets (27.1–32.7% range) exceeded that of full-fat soybean meal (19%) [44] and commercially available fishmeal (20.0–25.5% range) [45].

The dietary and larval fat contents were positively correlated. This suggests that larvae can accumulate body fat in proportion to dietary fat availability, as also shown by Nguyen et al. (2015) [11]. In that study, larvae reared on fruits and vegetables had the lowest fat (2.22%), and those reared on fish had the highest fat content (11.6%), which reflected the diet’s fat contents (1.55% and 36.18%, respectively).

The larval fatty acid (FA) composition also differed between diets. FF-based diets produced higher larval fatty acids than PS-based diets. The larval FA profile also varied, reflecting the diet’s fat content. This agrees with the results of different studies, showing that the fatty acid composition is affected by the diet’s fat content [46,47,48]. Specifically, the sum of saturated fatty acids was higher in larvae reared on FF-based diets than on PS-based diets, in accordance with the literature [33,46]. In our study, the predominant larval fatty acids were lauric acid, palmitic acid, stearic acid, oleic acid, linoleic acid and myristic acid, broadly reflecting the dietary fatty acid profile. These results align with Shumo et al. (2019), although their values were relatively higher, except for lauric acid [47].

Beyond the nutritional composition and particularly the crude protein of substrates, other factors such as free water and substrate particle size may influence the BSF larval growth. Free water in the substrate can affect larval growth and development [49], but no free water was observed during the daily inspection of the crates. In the current study, the substrates were shredded into different particle sizes, which may have affected the BSFL growth and performance. There is no ideal particle size established for BSFL, and what matters most is the physical structure the particles create in the substrate (porosity, bulk density, water release) [38]. Therefore, further studies should be conducted to determine the optimal particle size for BSFL feed.

Frass is also an end product of BSF production and is used as a soil fertiliser and amendment [50]. Despite similar diets’ CP calculated from nitrogen (N), frass N differed significantly, with the highest value for FF-MS-SW (3.8%). We observed a negative correlation between frass N and the diet’s ash (τ = −0.75), indicating N-rich frass was poor in ash, though more research is needed, as the literature on this is limited.

The literature shows that the frass quality and composition are strongly impacted by the feed substrates utilised in the rearing process, and thus substrate-dependent [51,52,53,54]. This partially complies with our results. The frass fat was by far the highest on the FF diet, which had the highest fat content, and undetectable in the frass of PS-based diets, which had a low fat content. Moreover, PS-based frasses were rich in Ca and P as the PS-based diets, consistent with another study [33]. However, the frass fibre did not show a positive correlation with the dietary fibre; PS-based diets were fibre-rich, yet their frass did not have the highest fibre, in contrast to another study [33]. Compared to a wider list of waste streams, frass produced from pig manure did not exhibit a superior nutrient composition [54]. Depending on the diet nutrient composition, nutrient-rich frass may indicate incomplete diet bioconversion. This could occur due to excess nutrients (or a specific nutrient) or reduced nutrient availability due to the presence of fibre, leading to nutrient accumulation in the frass.

According to the literature, the average N composition in frass ranges between 3.14 and 3.4%DM, P between 1.5 and 2.9%DM, and K between 2.91 and 2.99%DM [51,53]. Except for PS-MS-PM frass, frass N in this study fell within the range. Only FF-PS and PS-MS-PM frass had P content within the range, and none had enough K content.

## 5. Conclusions

BSFL growth and nutritional composition are affected by multiple factors, including the experimental setup, environmental conditions, the physical and chemical properties of rearing substrates, harvesting age, and processing methods. Although crude protein (CP) in the diet is often presumed to be the most critical factor, our study intentionally controlled for CP to examine the influence of other dietary components on larval growth and performance.

Overall, the results of this study indicated that dietary macronutrients other than CP may have a critical effect on larval growth and composition. Particularly, total fat, fibre and carbohydrates in the diet affected the larval composition, and the larval fatty acid profile reflected the diet’s fatty acid profile. While we cannot conclude from this study how BSFL deposit each macronutrient independently, the findings showed that larval CP deposition is not determined solely by the dietary CP concentration.

It is also difficult to formulate a solid conclusion regarding the frass composition because the frass is mixed with the unconsumed substrate, so the composition may not be highly reliable and representative of the excreta alone.

These findings highlight the need for studies that independently manipulate macronutrient levels and fatty-acid classes under controlled conditions, and that quantify the contribution of physical substrate properties (e.g., fibre type, particle size distribution, bulk density, porosity) to BSFL growth and composition.

## Figures and Tables

**Table 1:** Dry matter, crude protein and fat content (% on dry matter basis) of the different biowaste products.

Ingredient	Source	Dry Matter (%)	Crude Protein (% DM)	Fat (% DM)
Fast food (FF)	McDonald’s restaurants (The Netherlands)	55.0	20.4	27.0
Pig manure solid (PS)	Van Beek SPF Varkens B.V. (Lelystad, The Netherlands)	33.0	18.2	6.0
Mushroom stems (MS) *Agaricus bisporus*	CNC Grondstoffen BV (Milsbeek, The Netherlands)	10.0	28.4	0.0
Secondary sludge from slaughter waste (SW)	Esbro (Doetinchem, The Netherlands)	42.0	36.9	27.0
Commercial poultry meal (PM)	Sonac Burgum B.V. (Burgum, The Netherlands)	96.0	72.9	13.0
Cellulose	VWR International (Amsterdam, The Netherlands)	100.0	0.0	0.0

**Table 2:** Composition of the different diets given in kg on a fresh matter (FM) basis and in percentages of the total ingredient contribution on a dry matter (DM) basis.

	Diet											
	FF		FF-PS		FF-MS-PM		FF-MS-SW		PS-MS-PM		PS-MS-SW	
Ingredient	FM (kg)	DM (%)	FM (kg)	DM (%)	FM (kg)	DM (%)	FM (kg)	DM (%)	FM (kg)	DM (%)	FM (kg)	DM (%)
Fast food (FF)	27.2	92.0	10.7	49.0	10.7	49.0	10.8	49.0				
Pig manure solid (PS)			18.1	49.7					19.1	52.0	19.2	52.0
Mushroom stem (MS)					16.8	14.0	17.0	14.0	12.8	10.5	12.8	10.5
Slaughter waste (SW)							4.7	16.3			5.59	20.5
Poultry meal (PM)			0.2	1.3	1.0	8.2			1.3	10.4		
Cellulose	1.3	8			3.5	28.8	2.5	20.7	3.3	27.1	2.1	17.0
Water	23.4		11.1		8.0		5.4		4.0		0.6	
TOTAL	51.9	100.0	40.1	100.0	40.0	100.0	40.4	100.0	40.5	100.0	40.6	100.0

**Table 3:** Biochemical composition and pH of the substrates (dry matter expressed in %, all macronutrients expressed in % of dry matter).

	FF	FF-PS	FF-MS-PM	FF-MS-SW	PS-MS-PM	PS-MS-SW	SEM	*p*-Value
Dry matter	30.83 ^a^	29.40 ^b^	29.67 ^ab^	29.10 ^bc^	28.17 ^c^	28.93 ^bc^	0.24	0.0003
Crude ash	3.68 ^e^	10.32 ^c^	5.28 ^d^	5.04 ^d^	12.54 ^a^	11.18 ^b^	0.14	<0.0001
Crude protein (N × 6.25)	21.71	21.54	22.46	24.51	20.83	20.85	0.78	0.0584
Total fat	31.23 ^a^	19.83 ^c^	20.55 ^c^	27.26 ^b^	5.32 ^e^	11.52 ^d^	0.4	<0.0001
Crude fibre	6.49 ^f^	12.81 ^e^	23.83 ^c^	18.22 ^d^	32.89 ^a^	27.76 ^b^	0.51	<0.0001
Starch	29.31 ^a^	12.14 ^b^	11.57 ^b^	11.56 ^b^	-	-	0.56	<0.0001
N-free substances	36.89 ^a^	35.49 ^a^	27.87 ^b^	24.98 ^b^	28.41 ^b^	28.69 ^b^	0.79	<0.0001
Potassium (K)	0.58 ^d^	0.71 ^c^	0.78 ^bc^	0.77 ^bc^	0.95 ^a^	0.78 ^b^	0.01	<0.0001
Calcium (Ca)	0.17 ^e^	1.93 ^c^	0.76 ^d^	0.72 ^d^	2.52 ^a^	2.28 ^b^	0.03	<0.0001
Phosphorus (P)	0.31 ^c^	1.03 ^b^	0.44 ^c^	0.41 ^c^	1.27 ^a^	1.09 ^ab^	0.04	<0.0001
pH	4.52 ^d^	5.51 ^c^	4.68 ^d^	4.54 ^d^	7.39 ^a^	6.38 ^b^	0.05	<0.0001

Means with different superscript letters differ significantly (two-way ANOVA followed by Tukey’s post hoc test analysis, *p* < 0.05). Fast food waste (FF), solid pig manure (PS), mushroom stems (MS), slaughter waste (SW) and poultry meal (PM). SEM: standard error of the mean.

**Table 4:** Fatty acid composition of the experimental diets (expressed in % on DM basis).

Fatty Acid (FA)	FF	FF-PS	FF-MS-PM	FF-MS-SW	PS-MS-PM	PS-MS-SW	SEM	*p*-Value
Capric acid (C 10:0)	0.03	0.03	0.04	0.05	-	-	0.01	0.4206
Lauric acid (C 12:0)	0.06 ^b^	0.09 ^b^	0.07 ^b^	0.24 ^a^	0.07 ^b^	0.22 ^a^	0.01	<0.0001
Myristic acid (C 14:0)	0.68 ^a^	0.52 ^b^	0.47 ^b^	0.63 ^a^	0.12 ^d^	0.27 ^c^	0.02	<0.0001
Palmitic acid (C 16:0)	5.81 ^a^	4.32 ^b^	3.97 ^b^	5.71 ^a^	1.47 ^d^	3.18 ^c^	0.11	<0.0001
Stearic acid (C 18:0)	2.85 ^a^	2.59 ^ab^	1.84 ^c^	2.32 ^b^	1.33 ^d^	1.62 ^cd^	0.07	<0.0001
Oleic acid C 18:1	14.24 ^a^	7.21 ^d^	8.87 ^c^	11.58 ^b^	0.63 ^f^	2.82 ^e^	0.21	<0.0001
Linoleic acid C 18:2 omega-6	4.15 ^a^	2.47 ^c^	2.92 ^b^	3.80 ^a^	0.43 ^e^	1.60 ^d^	0.08	<0.0001
Sum saturated FAs	9.91 ^a^	8.04 ^b^	6.72 ^c^	9.30 ^a^	3.28 ^e^	5.57 ^d^	0.19	<0.0001
Sum monounsaturated FAs	16.47 ^a^	8.90 ^d^	10.38 ^c^	13.65 ^b^	1.51 ^f^	4.09 ^e^	0.23	<0.0001
Sum polyunsaturated FAs	4.77 ^a^	2.85 ^d^	3.40 ^c^	4.24 ^b^	0.52 ^f^	1.82 ^e^	0.10	<0.0001
Sum trans FAs	0.50	0.49	0.30	0.27	0.46	0.30	0.06	0.0501
Omega-3 FAs	0.37 ^a^	0.22 ^c^	0.25 ^c^	0.30 ^b^	0.03 ^e^	0.10 ^d^	0.01	<0.0001
Omega-6 FAs	4.18 ^a^	2.48 ^c^	2.98 ^b^	3.86 ^a^	0.46 ^e^	1.66 ^d^	0.08	<0.0001

Means with different superscript letters differ significantly (two-way ANOVA followed by Tukey’s post hoc test analysis, *p* < 0.05). Fast food waste (FF), solid pig manure (PS), mushroom stems (MS), slaughter waste (SW) and poultry meal (PM).

**Table 5:** Fatty acid content of black soldier fly larvae reared on different diets (expressed in % on DM basis).

Fatty Acid (FA)	FF	FF-PS	FF-MS-PM	FF-MS-SW	PS-MS-PM	PS-MS-SW	SEM	*p*-Value
Capric acid (C 10:0)	0.54 ^a^	0.26 ^b^	0.18 ^c^	0.21 ^bc^	0.04 ^d^	0.07 ^d^	0.01	<0.0001
Lauric acid (C 12:0)	6.04 ^c^	9.81 ^a^	7.70 ^bc^	9.05 ^ab^	1.18 ^d^	2.21 ^d^	0.36	<0.0001
Myristic acid (C 14:0)	1.37 ^b^	1.86 ^a^	1.59 ^ab^	1.71 ^ab^	0.39 ^c^	0.74 ^c^	0.07	<0.0001
Palmitic acid (C 16:0)	4.98 ^a^	5.71 ^a^	4.50 ^a^	4.97 ^a^	2.04 ^b^	4.49 ^a^	0.27	<0.0001
Stearic acid (C 18:0)	1.40 ^a^	1.05 ^b^	0.81 ^b^	0.75 ^bc^	0.50 ^c^	0.97 ^b^	0.06	<0.0001
Oleic acid C 18:1	9.47 ^a^	7.97 ^ab^	7.07 ^b^	7.19 ^b^	1.84 ^d^	4.62 ^c^	0.31	<0.0001
Linoleic acid C 18:2 omega-6	3.80 ^a^	3.73 ^a^	3.43 ^a^	3.96 ^a^	0.80 ^c^	1.72 ^b^	0.16	<0.0001
Sum saturated FAs	14.53 ^b^	18.96 ^a^	14.94 ^b^	16.86 ^ab^	4.38 ^d^	8.79 ^c^	0.73	<0.0001
Sum monounsaturated FAs	11.29 ^a^	9.49 ^ab^	8.18 ^bc^	8.52 ^b^	2.85 ^d^	6.17 ^c^	0.42	<0.0001
Sum polyunsaturated FAs	4.34	4.18	3.91	4.50	2.72	1.93	0.67	0.1147
Sum trans FAs	0.12	0.13	0.04	NA	1.97	0.25	0.79	0.4306
Omega-3 FAs	0.41 ^a^	0.39 ^a^	0.38 ^a^	0.45 ^a^	0.06 ^b^	0.13 ^b^	0.03	<0.0001
Omega-6 FAs	3.87 ^a^	3.79 ^a^	3.51 ^a^	4.04 ^a^	0.85 ^c^	1.80 ^b^	0.17	<0.0001

Means with different superscript letters differ significantly (two-way ANOVA followed by Tukey’s post hoc test analysis, *p* < 0.05). Fast food (FF), pig manure solid (PS), mushroom stems (MS), slaughter waste (SW) and poultry meal (PM).

**Figure A1:**
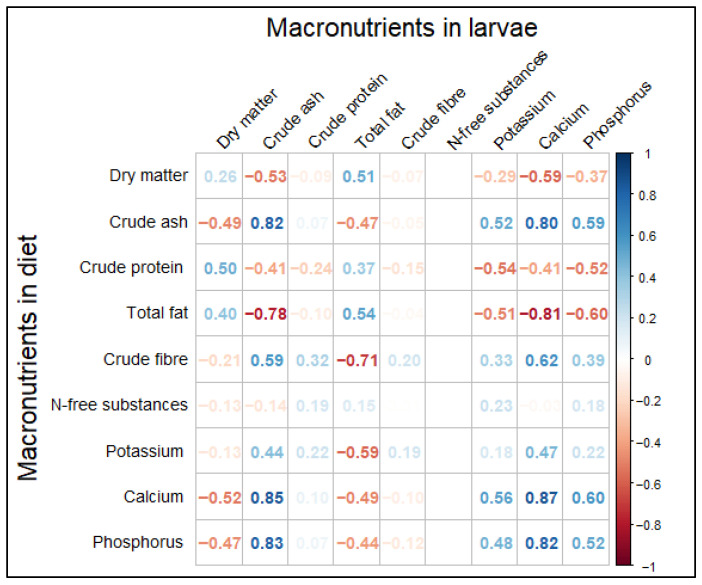
Correlation analysis of the biochemical composition of the diets and the larvae.

**Figure A2:**
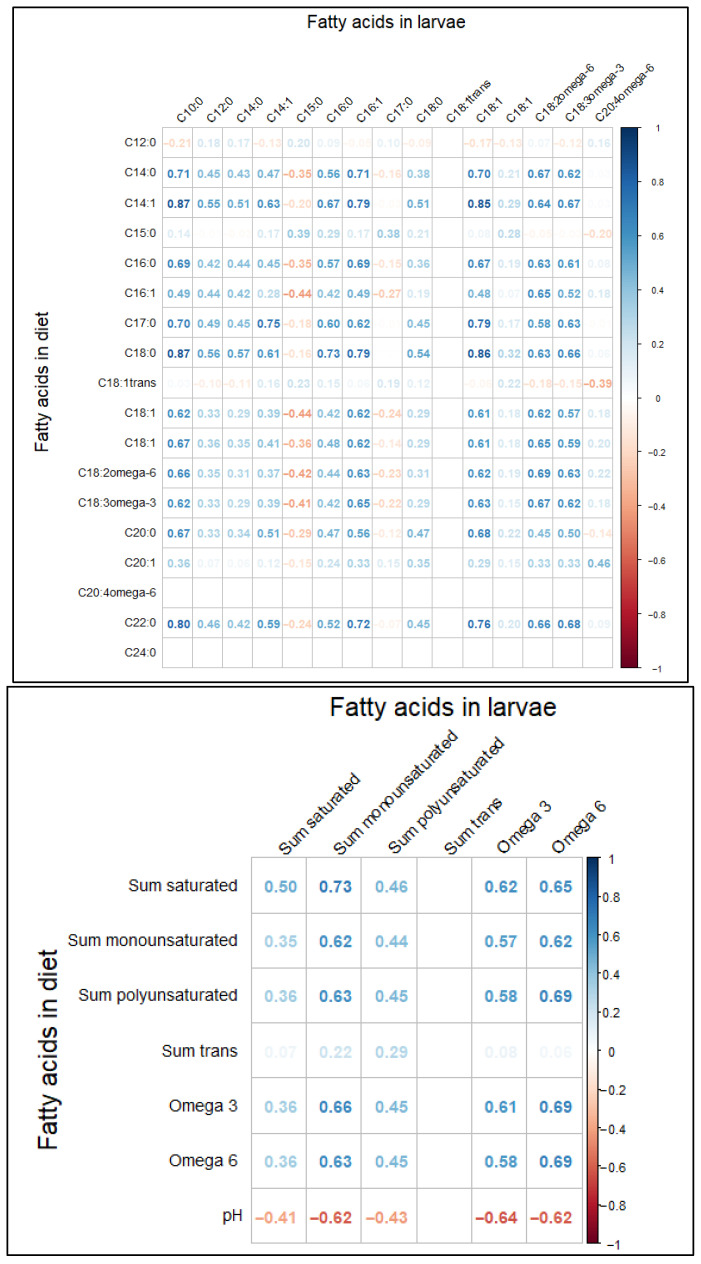
Correlation analysis of the fatty acid profile composition of the diets and the larvae.

## Data Availability

The original contributions presented in this study are included in the article. Further inquiries can be directed to the corresponding author.

## Abbreviations

The following abbreviations are used in this manuscript:

BSF	Black Soldier Fly
BSFL	Black Soldier Fly Larvae

## Appendix A

**Table A1:** Fatty acid composition of the experimental diets (expressed as % of dry matter).

Fatty Acid	FF	FF-PS	FF-MS-PM	FF-MS-SW	PS-MS-PM	PS-MS-SW	SEM	*p*-Value
Myristoleic acid (C 14:1)	0.25 ^a^	0.22 ^b^	0.16 ^d^	0.19 ^c^	0.09 ^e^	0.10 ^e^	0.004	<0.0001
Pentadecanoic acid (C 15:0)	0.09 ^b^	0.14 ^a^	0.06 ^c^	0.08 ^bc^	0.10 ^b^	0.10 ^b^	0.01	<0.0001
Palmitoleinic acid (C 16:1)	0.93 ^a^	0.60 ^b^	0.65 ^b^	0.99 ^a^	0.15 ^d^	0.48 ^c^	0.02	<0.0001
Margaric acid (C 17:0)	0.16 ^a^	0.16 ^a^	0.11 ^b^	0.11 ^b^	0.07 ^c^	0.07 ^c^	0.01	<0.0001
Stearic acid (C 18:0)	2.85 ^a^	2.59 ^ab^	1.84 ^c^	2.32 ^b^	1.33 ^d^	1.62 ^cd^	0.07	<0.0001
Octadecenoic acid trans-isomers C 18:1 trans	0.29 ^ab^	0.34 ^ab^	0.14 ^b^	0.19 ^b^	0.42 ^a^	0.24 ^ab^	0.04	0.0118
Oleic acid C 18:1	14.24 ^a^	7.21 ^d^	8.87 ^c^	11.58 ^b^	0.63 ^f^	2.82 ^e^	0.21	<0.0001
cis-vaccenic acid C 18:1	0.58 ^a^	0.40 ^b^	0.40 ^b^	0.55 ^a^	0.14 ^d^	0.25 ^c^	0.01	<0.0001
Octadecadienoic acid trans-isomers C 18:2 trans	0.17 ^a^	0.13 ^a^	0.11 ^a^	-	0.02 ^b^	-	0.01	0.0011
Linoleic acid C 18:2 omega-6	4.15 ^a^	2.47 ^c^	2.92 ^b^	3.80 ^a^	0.43 ^e^	1.60 ^d^	0.08	<0.0001
Octadecatetrienic acid, trans-isomers C 18:3 trans	0.04	-	0.03	-	-	-	0.01	0.5363
alpha-linolenic acid C 18:3 omega-3	0.37 ^a^	0.22 ^c^	0.25 ^c^	0.30 ^b^	0.03 ^e^	0.10 ^d^	0.01	<0.0001
Arachic acid C 20:0	0.09 ^a^	0.08 ^ab^	0.06 ^bc^	0.06 ^bc^	0.05 ^c^	0.05 ^c^	0.005	0.0002
Eicosenoic acid C 20:1	0.18	0.13	0.14	0.15	0.08	0.17	0.03	0.3819
Arachidonic acid C 20:4 omega-6	0.03 ^bc^	-	0.05 ^ab^	0.05 ^a^	0.02 ^c^	0.04 ^ac^	0.004	0.0062
Behenic acid C 22:0	0.10 ^a^	0.08 ^ab^	0.06 ^bc^	0.07 ^ab^	0.03 ^cd^	0.03 ^d^	0.01	<0.0001
Lignoceric acid C 24:0	0.03	0.04	0.03	0.03	0.03	-	0.004	0.2690

Means with different superscript letters differ significantly (two-way ANOVA followed by Tukey’s post hoc test analysis, *p* < 0.05). Fast food waste (FF), solid pig manure (PS), mushroom stems (MS), slaughter waste (SW) and poultry meal (PM).

**Table A2:** Fatty acid content of BSFL reared on different diets (expressed as % of dry matter).

Fatty Acid	FF	FF-PS	FF-MS-PM	FF-MS-SW	PS-MS-PM	PS-MS-SW	SEM	*p*-Value
Myristoleic acid (C 14:1)	0.15 ^ab^	0.17 ^a^	0.12 ^bc^	0.12 ^ac^	0.09 ^c^	0.08 ^c^	0.01	0.0009
Pentadecanoic acid (C 15:0)	0.06 ^b^	0.16 ^a^	0.08 ^b^	0.07 ^b^	0.12 ^ab^	0.16 ^a^	0.01	0.0007
Hexadecanoic acid trans-isomers C 16:1 trans	-	-	-	-	0.04	0.06	0.01	0.5517
Palmitoleinic acid (C 16:1)	1.22 ^a^	0.97 ^ab^	0.77 ^bc^	0.93 ^ab^	0.56 ^c^	0.78 ^bc^	0.06	0.0005
Margaric acid (C 17:0)	0.09	0.11	0.08	0.07	0.08	0.12	0.01	0.0802
Octadecenoic acid trans-isomers C 18:1 trans	0.08 ^b^	0.12 ^ab^	0.04 ^b^	-	0.11 ^ab^	0.19 ^a^	0.02	0.0056
cis-vaccenic acid C 18:1	0.27 ^a^	0.16 ^b^	0.13 ^b^	0.14 ^b^	0.13 ^b^	0.18 ^b^	0.02	0.0019
alpha-linolenic acid C 18:3 omega-3	0.37 ^a^	0.36 ^a^	0.33 ^a^	0.35 ^a^	0.06 ^b^	0.10b	0.01	<0.0001
Arachic acid C 20:0	0.03	-	-	-	-	0.03	0.005	0.7832
Eicosenoic acid C 20:1	0.10	-	-	0.10	-	0.26	0.07	0.2831
Arachidonic acid C 20:4 omega-6	0.07	0.07	0.08	0.08	0.04	0.08	0.01	0.0878
Eicosapentaenic acid C 20:5 omega-3	0.04	NA	0.04	0.07	NA	NA	0.01	0.2591

Means with different superscript letters differ significantly (two-way ANOVA followed by Tukey’s post hoc test analysis, *p* < 0.05). Fast food (FF), pig manure solid (PS), mushroom stems (MS), slaughter waste (SW) and poultry meal (PM).
